# Green synthesis and characterization of binary, ternary, and quaternary Ti/MMO anodes for chlorine and oxygen evolution reactions

**DOI:** 10.1038/s41598-024-59595-2

**Published:** 2024-04-29

**Authors:** A. B. Abdel-Aziz, F. El-Taib Heakal, R. M. El Nashar, I. M. Ghayad

**Affiliations:** 1 October High Institute for Engineering & Technology, Giza, 12596 Egypt; 2 Chemistry Department, Faculty of Science, Cairo University, Giza, 12613 Egypt; 3 Central Metallurgical Research and Development Institute (CMRDI), Cairo, 12422 Egypt

**Keywords:** MMO, RuO_2_, TiO_2_, IrO_2_, and Ta_2_O_5_, Stability test, Chlorine evolution reaction (CER), Oxygen evolution reaction (OER), Chemistry, Materials science

## Abstract

Dimensionally stable anodes of titanium (Ti) metal coated with mixed metal oxides (MMO) are widely used in several electrochemical applications, especially chloro-alkali electrolysis. Herein, we deposited MMO coatings on Ti substrates in different compositions, namely, (60%RuO_2_-40%TiO_2_), (60%RuO_2_-30%TiO_2_-10%IrO_2_), and (60%RuO_2_-20%TiO_2_-15%IrO_2_-5%Ta_2_O_5_), where RuO_2_ has the same percentage ratio in all coatings. The aim was to use these electrodes for chlorine evolution reaction (CER) and oxygen evolution reaction (OER) applications. Electrochemical characterization of the coated samples was performed to identify the best Ti/MMO electrodes with the highest efficiencies among the various prepared combinations. The role of IrO_2_ and Ta_2_O_5_ in enhancing corrosion resistance and electrochemical efficacy was up for debate. Scanning electron microscope (SEM), energy dispersive spectroscopy (EDS), X-ray diffraction (XRD), and X-ray photoelectron spectroscopy (XPS) analyses were exploited to determine the surface morphology, chemical composition, crystallinity, surface composition, and chemical states of the acquired coatings. The differential scanning calorimetry (DSC) method was used to evaluate the apparent activation energy (${{\text{E}}}_{{\text{a}}}$) of the deposited MMO. Additionally, the electrochemical performance of our designed coatings was scrutinized by cyclic voltammetry (CV), electrochemical impedance spectroscopy (EIS), a current on–off test, a CV stability test (ST), and an accelerated stability test (AST). Furthermore, linear sweep voltammetry (LSV) was incorporated to assess the catalytic efficacy of the prepared anodes toward the CER in a brine solution of pH 2 and the OER in 1 M H_2_SO_4_. It became clear that the CER and OER incurred almost the same potential value (1.1 V) on both Ti/RuO_2_-TiO_2_ and Ti/RuO_2_-TiO_2_-IrO_2_ electrodes. However, on the Ti/RuO_2_-TiO_2_-IrO_2_-Ta_2_O_5_ anode, there was a 0.2 V potential difference between the CER occurring at 1.1 V and the OER happening at 1.3 V.

## Introduction

Electrolysis of a concentrated sodium chloride (brine) solution, which produces chlorine gas and sodium hydroxide, is one of the most common industrial electrolytic processes worldwide^1^. Chlorine is essential for water disinfection and is used to assemble polyvinyl chloride (PVC), as well as in the production of a variety of other chlorinated polymers, solvents, and specialized compounds^2^. In the meantime, sodium hydroxide is widely used in the pulp and paper industry, the extraction of aluminum from bauxite, and the manufacture of chemicals^3^.

Ti/MMO anodes are widely used in the chlor-alkali industry due to their excellent electrocatalytic activity for the chlorine evolution reaction (CER) and their chemical and mechanical stability^4^. The Ti/MMO anode typically consists of a Ti metal substrate covered with low-overpotential noble metal oxides and their blend with titanium dioxide (TiO_2_). The most common binary oxide mixture is RuO_2_-TiO_2_. The thermal breakdown of RuCl_3_.3H_2_O and TiCl_3_ in an organic solvent is the most popular preparation technique for such coatings^5–7^.

RuO_2_-based materials have recently received attention due to their high conductivity, excellent thermal stability, and high resistance to chemical corrosion^8^. Anodes composed of RuO_2_ demonstrate high electrocatalytic activity for CER in acidic conditions. However, when oxygen evolved, these coatings were recognized to be susceptible to corrosion. The CER and OER occur concurrently due to a low difference (0.13 V) in their standard potentials. The standard potential of both CER and OER (versus the standard hydrogen electrode (SHE)) is 1.36 V and 1.23 V, respectively. In moderately acidic conditions (pH < 4.0), CER is preferable over OER. When the pH of the medium increases, the selectivity towards OER rises, while it decreases for CER^9^.

Anodic coatings for OER are commonly composed of RuO_2_, IrO_2_, and Ta_2_O_5_. Furthermore, the actual surface area and the number of active sites linked with the anode activity suggest that stable and active catalysts with a large accessible surface area can reduce the overpotential of both processes^10,11^. Valve metals, such as titanium and tantalum, allow electricity to flow only in one direction. In a galvanic cell, they can typically serve as a cathode and not an anode, owing to the production of a very resistive thin oxide film on their surfaces under anodic conditions. Moreover, the corrosion resistance of Ta implants was reported to be significantly greater than that of Ti implants due to the spontaneous development of a thick Ta_2_O_5_ protective coating on exposure to air^12^.

In the chlor-alkali industry, the main effective chemical reactions are the cathodic hydrogen evolution reaction (HER) and the anodic chlorine evolution reaction (CER), as presented in the following two Eqs. (1) and (2), respectively^13^:

1 $${\text{2H}}_{{2}} {\text{O }} + {\text{ 2e}}^{ - } \to {\text{ H}}_{{2}} \uparrow \, + {\text{ 2OH}}^{ - } \,\,\,\,\,\,{\text{E}}^{^\circ } = \, - 0.{\text{83 V }}\left( {{\text{vs}}.{\text{ SHE}}} \right)$$

2 $${\text{2Cl}}^{ - } \to {\text{ Cl}}_{{2}} \uparrow \, + {\text{ 2e}}^{ - }\,\,\,\,\,\, {\text{E}}^{^\circ } = { 1}.{\text{36 V }}\left( {{\text{vs}}.{\text{ SHE}}} \right)$$

Thus, the net reaction can be represented by Eq. (3)^14^:

3 $${\text{2Cl}}^{ - } + {\text{ 2H}}_{{2}} {\text{O }} \to {\text{ H}}_{{2}} \uparrow \, + {\text{ Cl}}_{{2}} \uparrow \, + {\text{ 2OH}}^{ - }$$

The CER takes place in two stages. In the first one, the chloride ion adsorbs on the surface-active site of the substrate (S), such as RuO_2,_ and oxidizes according to Eq. (4). After that, the adsorbed chloride ion interacts with a dissolved chloride ion or with another adsorbed chloride ion to produce chlorine gas, as given by Eqs. (5) and (6), respectively^15^:

4 $${\text{S }} + {\text{ Cl}}^{ - } \to {\text{ S}} - {\text{Cl}}_{{{\text{ads}}}} + {\text{ e}}^{ - }$$

5 $${\text{S}} - {\text{Cl}}_{{{\text{ads}}}} + {\text{ Cl}}^{ - } \to {\text{ S }} + {\text{ Cl}}_{{2}} \uparrow \, + {\text{ e}}^{ - }$$

6 $${\text{2S}} - {\text{Cl}}_{{{\text{ads}}}} \to {\text{ 2S }} + {\text{ Cl}}_{{2}} \uparrow$$

At high pH values, where the OER occurs, RuO_2_ is oxidized to the more soluble ruthenium oxide compound (RuO_4_), which leaches from the electrode surface^16^. In the chlor-alkali process, the concentration of chloride ions decreases with time, allowing the OER to compete with the CER. Degrading water in acidic solutions provides an example to analyze the OER. This process involves several chemical steps on the surface-active sites, as given by Eqs. (7), (8), (9), and (10)^17^. The OER rate may be determined by Eq. (8) for "compact" morphologies or Eq. (9) for "cracked" morphologies.

7 $${\text{S }} + {\text{ H}}_{{2}} {\text{O }} \to {\text{ S}} - {\text{OH }} + {\text{ H}}^{ + } + {\text{ e}}^{ - }$$

8 $${\text{S}} - {\text{OH }} \to {\text{ S}} - {\text{O }} + {\text{ H}}^{ + } + {\text{ e}}^{ - }$$

9 $${\text{2S}} - {\text{OH }} \to {\text{ S}} - {\text{O }} + {\text{ S }} + {\text{ H}}_{{2}} {\text{O}}$$

10 $${\text{2S}} - {\text{O }} \to {\text{ 2S }} + {\text{ O}}_{{2}} \uparrow \,\,\,\,\,\,\,{\text{E}}^\circ \, = { 1}.{\text{23 V }}\left( {{\text{vs}}.{\text{ SHE}}} \right)$$

Traditionally, most of the applications were done on binary MMO anodes. Herein, we have successfully synthesized the binary (RuO_2_-TiO_2_), ternary (RuO_2_-TiO_2_-IrO_2_), and quaternary (RuO_2_-TiO_2_-IrO_2_- Ta_2_O_5_) MMO coatings on Ti substrate via a facile green approach. Each metal oxide was introduced into the coating formulation to serve a specific role. In this way, RuO_2_ played the most essential role in electrocatalytic performance, while IrO_2_ was used to shift the OER potential far away from the CER potential, and Ta_2_O_5_ was combined with TiO_2_ to increase the corrosion resistance of the prepared electrodes. Non-toxic and green solvents, such as water with the addition of hydrochloric acid, were employed to avoid more toxic and expensive alkoxides or organic solvents used in the sol–gel method, aiming our simple dip coating technique to be more economically and eco-friendly. The coatings were characterized by different physical techniques, and the apparent activation energies of their thermal behavior were estimated based on the Kissinger method^18^. The electrocatalytic performance of the designed Ti/MMO anodes for brine electrolysis under conditions of OER was investigated and compared. Their electrocatalytic and enhancement efficiencies toward CER and OER were explored.

## Experimental

### Materials and pretreatment

Ruthenium trichloride trihydrate (RuCl_3_.3H_2_O), titanium trichloride (TiCl_3_), diammonium iridium hexachloride [(NH_4_)_2_IrCl_6_], and tantalum pentachloride (TaCl_5_) were all A.R. grade (Sigma-Aldrich) and used without further purification to prepare the coating baths. Titanium disc-shaped specimens with a 2 mm height thickness were prepared from a 20 mm radius titanium rod using a wire-cutting machine. The Ti substrates were mechanically sanded with coarse sandpaper with a roughness of 220 grains of sand to obtain a reasonably rough surface to aid the adhesion of the MMO coating. The polished samples were then sonicated in acetone for 10 min, pickled in boiling 33% hydrochloric acid (HCl) for 20 min, etched in 5% hydrofluoric acid (HF) for 5 min, washed with distilled water several times, and finally dried at 80 °C. The weight of the activated samples was then estimated using a 4-digit analytical balance before applying the coating process.

### Synthesis of Ti/MMO electrodes

Each activated sample was coated by the simple dip coating technique in the respective precursor bath for a sufficient time of 2–3 s, dried at 130 °C for 20 min, later at 250 °C for 10 min to improve the adherence of the coating, and finally calcinated at 450 °C for 10 min to eliminate all chloride ions and form the oxides. The coating procedure was applied 5 and 10 times (cycles) to obtain the desired weight gain (~ 1.5 mg/cm^2^). Proper concentrations over a domain of 0.01 M to 0.5 M from the precursor (RuCl_3_.3H_2_O and TiCl_3_) solution were prepared in 1:1 of 33% HCl and distilled water. As depicted in Table 1, seven binary samples (B1 to B7) with 60%RuO_2_-40%TiO_2_ were prepared. Based on the appropriate concentration of the binary coating (B3), a ternary sample (B8) was also prepared by replacing 10% of TiCl_3_ in the deposition bath with (NH_4_)_2_IrCl_6_ precursor at the specified concentration. This was attempted to move the OER potential far away from the CER potential. Thereafter, by replacing another 10% of TiCl_3_ with both (NH_4_)_2_IrCl_6_ and TaCl_5_ to obtain the quaternary sample (B9) with 15% (NH_4_)_2_IrCl_6_ and 5% TaCl_5_ in the deposition bath. This was intended to boost the corrosion resistance of the synthesized electrode. To validate the added 5% Ta_2_O_5_ ratio to prepare the quaternary B9 electrode, another quaternary electrode containing 10% Ta_2_O_5_ was also prepared and compared with the first one. As shown in Fig. S1, it is evident that increasing the Ta content in the synthesized MMO electrode leads to an unwanted significant reduction in its efficiency. Therefore, 5% Ta_2_O_5_ was considered the optimum ratio of the quaternary coating necessary for the present application.

**Table 1:** Bath composition of the different MMO-coated samples.

Bath for sample	Molar Conc. of Precursor	Content of RuCl_3_.3H_2_O (g/100 mL)	Content of [(NH_4_)_2_IrCl_6_] (g/100 mL)	Content of TaCl_5_ (g/100 mL)	Content of TiCl_3_ (g/100 mL)	Wt.% of proposed MMO
B1	0.01	0.1569	–	–	0.0617	60%RuO_2_-40%TiO_2_
B2	0.05	0.7847	–	–	0.3085	
B3	0.1	1.5694	–	–	0.6169	
B4	0.2	3.1388	–	–	1.2338	
B5	0.3	4.7083	–	–	1.8507	
B6	0.4	6.2777	–	–	2.4676	
B7	0.5	7.8471	–	–	3.0845	
B8	0.1	1.5694	0.4410	–	0.4627	60%RuO_2_-30%TiO_2_-10%IrO_2_
B9	0.1	1.5694	0.6615	0.1791	0.3085	60%RuO_2_-20%TiO_2_-15%IrO_2_-5% Ta_2_O_5_

### Physical characterization techniques

Surface characterization of the MMO-coated samples was performed using a field emission scanning electron microscope (FESEM Quanta FEG 250). The Thermo Fisher Quanta FEG 250 can be operated under 1.2 nm at 30 kV and 2.3 nm at 1 kV with beam deceleration in high vacuum using the in-column detector. The Quanta FEG 250 is equipped with an energy-dispersive X-ray spectrometer (EDS) unit with a field emission gun operating at 20 kV. It can provide rapid, qualitative, and quantitative elemental composition analysis with a sample depth range of about 1–4 microns.

The prepared Ti/MMO electrodes were also analyzed using X-ray diffractometer D8 Discover from Bruker, which was operated at 40 kV and 40 mA, model X’Pert PRO, with a source Cu 1.54 Å K_α_ radiation, to determine the exact phases of the formed MMO. K-Alpha X-ray photoelectron spectrometer (XPS) system was used to deliver both elemental and chemical state information for Ti/MMO electrodes. It is an extremely surface-selective technique; the data is collected from the outer 10 nm of the sample and a critical surface area of about 40 nm.

### Thermal characterization techniques

The coated layers were separated from the prepared samples and their thermal properties were examined using simultaneous differential scanning calorimetry (DSC) and thermogravimetry (TG) analyses. The kinetic apparent activation energy (${{\text{E}}}_{{\text{a}}}$) was then calculated via the Kissinger method^19^. Experiments were performed in an aluminum crucible using a 10 mg powder sample of the separated coating layers at various heating rates, namely, 5, 10, 15, and 20 ℃/min over the temperature range from room temperature up to 1000 °C. During the test, high-purity argon gas (99.99%) was purged continuously at a flow rate of 60 mL/min.

### Electrochemical characterization techniques

Electrochemical properties of the various prepared Ti/MMO electrodes were investigated using cyclic voltammetry (CV), electrochemical impedance spectroscopy (EIS), current on–off test, cyclic voltammetry stability test (ST), and accelerated stability test (AST). The performance of the prepared anodes toward the chlorine evolution reaction (CER) and the oxygen evolution reaction (OER) was investigated using linear sweep voltammetry (LSV). All electrochemical characterizations were conducted using Autolab potentiostat/galvanostat (PGSTAT) driven with NOVA Autolab 1.8 software packages. The tests were executed in a conventional three-electrode cell using the prepared Ti/MMO coated sample as the working electrode, a square platinum sheet of size 5 mm × 5 mm × 1 mm as the counter electrode, and a saturated calomel electrode (SCE) as the reference electrode. EIS measurements were recorded by applying an AC sinusoidal perturbation signal with 10 mV amplitude over a large frequency domain from 100 kHz down to 50 mHz. Cyclic and linear sweep voltammetry measurements were carried out over the potential window of 0–1.2 V (vs. SCE) for the CV and 0–2.5 V (vs. SCE) for the LSV.

## Results and discussion

**Figure 1:**
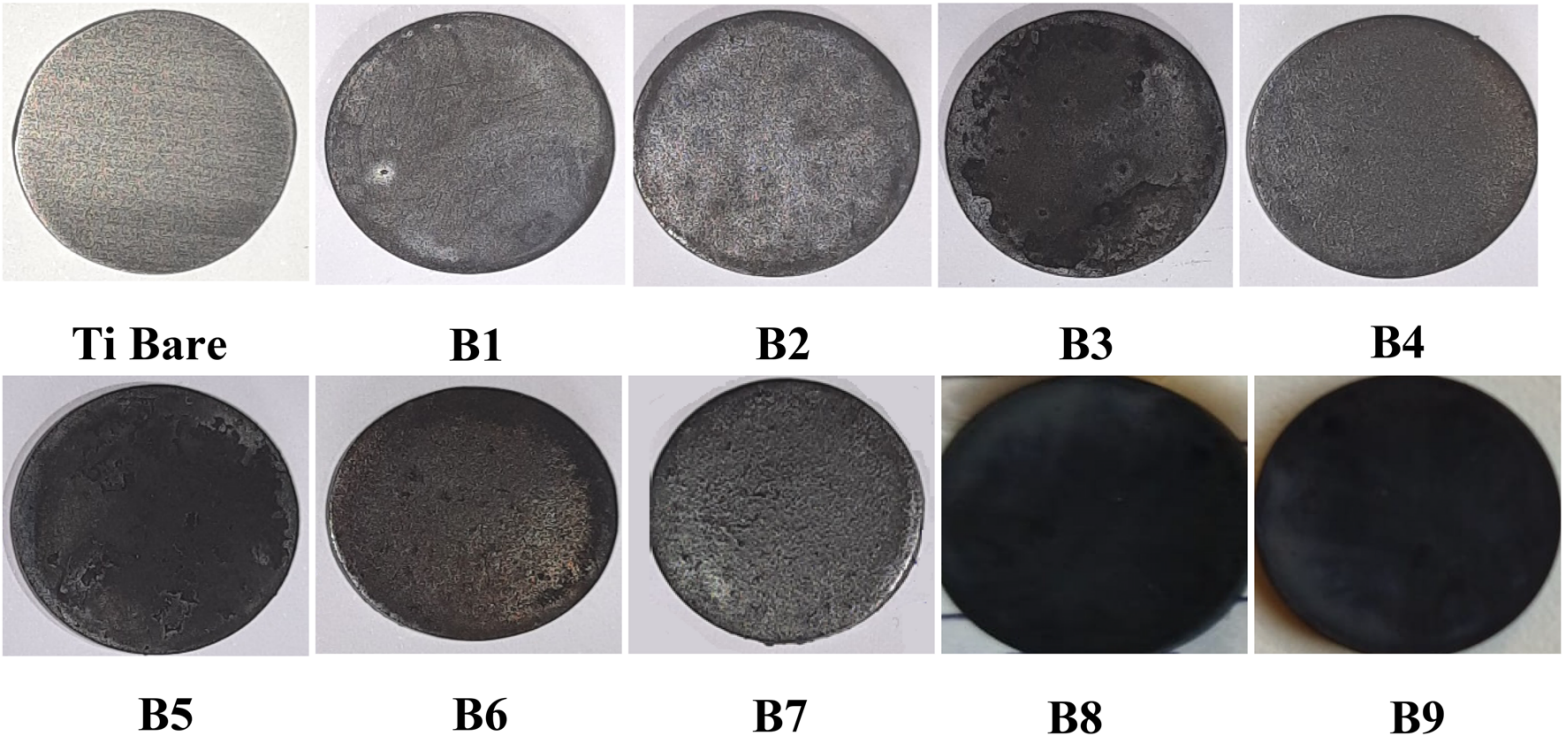
Photographs of the prepared (B1–B9) Ti/MMO samples.

The coated samples are photographed using a 48-megapixel phone camera, as seen in Fig. 1. Each sample, numbered B1 through B7, with a proposed 60%RuO_2_-40%TiO_2_ MMO composition, has a black appearance. As the concentration of the precursor increased, the color became denser and darker. After 10 deposition cycles, the highest precursor concentration of 0.5 M (sample B7) obtained the maximum weight gain of 7.23 mg/cm^2^ as MMO coating on the Ti substrate surface. Meanwhile, samples B8 and B9 were coated by denser MMO layers with weight gains of 1.87 and 1.95 mg/cm^2^ constituted 60%RuO_2_-30%TiO_2_-10%IrO_2_ and 60%RuO_2_-20%TiO_2_-15%IrO_2_-5% Ta_2_O_5_ MMO, respectively. Compared to sample B3 of the 60%RuO_2_-40%TiO_2_ composition with the same precursor concentration, which showed a weight gain of 1.59 mg/cm^2^. The photos in Fig. 1 indicate that the sample surfaces are completely covered by black coatings. As opposed to this, an uncoated Ti substrate should be blue due to the oxidized TiO_2_.

Table 2 displays the weight gain of the coated samples after 5 and 10 coating cycles. As can be seen, the weight gain of the MMO coating increases as the precursor concentration or the number of deposition cycles increases. Therefore, the coating cycle was repeated 10 times in the following experiments to reach the desired weight gain at relatively lower precursor concentrations of 0.1 M. This was used as the standard concentration to make the ternary or quaternary MMO coatings for B8 and B9 samples, respectively.

**Table 2:** Weight gain of the MMO-coated samples after 5- and 10-coating cycles.

Sample	Weight gain after 5-coating cycles (mg/cm^2^)	Weight gain after 10-coating cycles (mg/cm^2^)
B1	0.16	0.19
B2	0.32	0.48
B3	1.24	1.59
B4	1.72	2.83
B5	2.48	5.51
B6	2.77	6.30
B7	2.99	7.23
B8	1.15	1.87
B9	1.33	1.95

### Physical characterization

**Figure 2:**
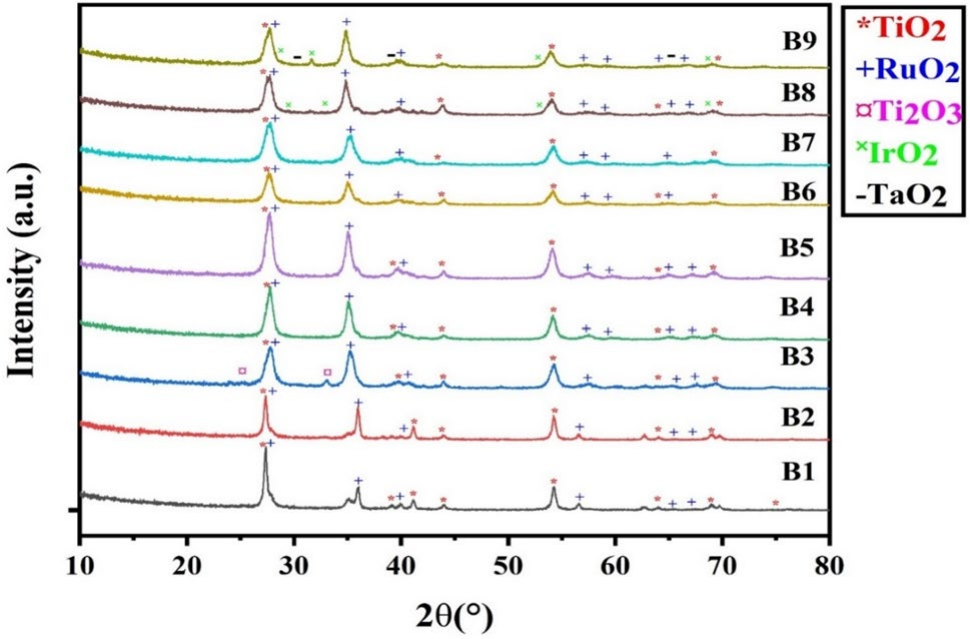
XRD patterns of the prepared (B1–B9) MMO samples.

The crystal phases of the prepared MMO samples were explored using X-ray diffraction (XRD) analysis. As presented in Fig. 2**,** the sharp peaks of the synthesized coatings affirm the crystalline nature of the produced MMO layers. The main peaks of TiO_2_ and RuO_2_ for all prepared samples appeared at the following 2Theta angles of (27.72°, 39.78°, 44.01°, 54.08°, 65.06°, and 69.32°)^20^ and (27.72°, 35.23°, 40.02°, 57.43°, 59.85°, 65.06°, and 67.15°)^21^, respectively. Additional IrO_2_ and Ta_2_O_5_ peaks are detected for B8 and B9 samples at 2Theta angles of (27.79°, 34.83°, 53.93°, and 69.26°)^22^ and (31.61°, 39.90°, and 66.82°)^23^, respectively. The peak list yields tetragonal space groups for titania (TiO_2_), ruthenium oxide (RuO_2_), iridium oxide (IrO_2_), and tantalum oxide (Ta_2_O_5_). The absence of diffraction peaks for the non-oxidized metallic states Ru, Ir, or Ta materials signifies that the coatings have been successfully heat-treated and completely transformed to their corresponding crystalline oxide phases. It is noteworthy to mention that the intensities of the RuO_2_ diffraction peaks are lower for the B1 and B2 samples than those for the other prepared B3–B7 samples. That might be due to the lower concentration of Ru precursor ions in the coating baths for those samples.

**Figure 3:**
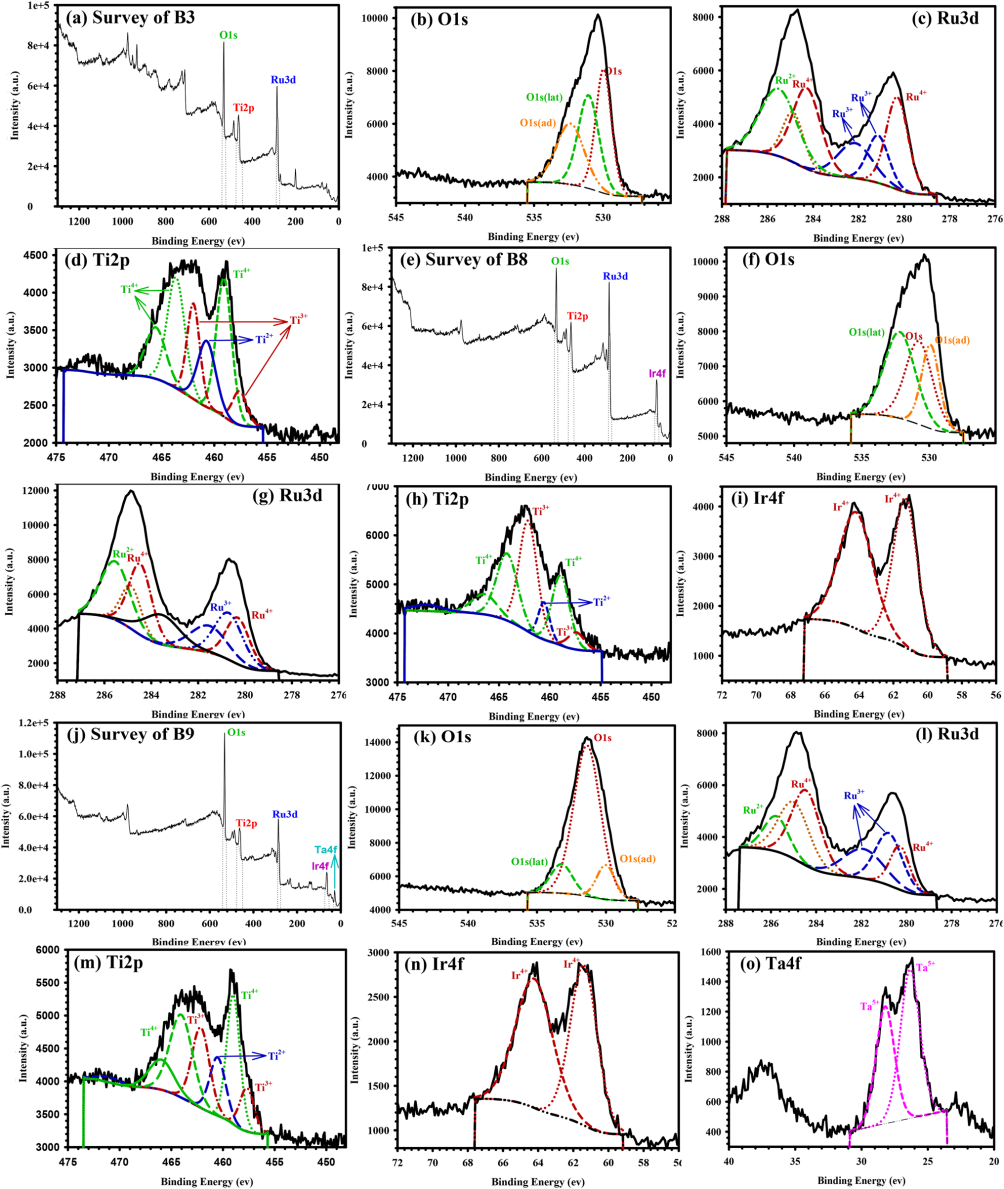
XPS Spectra of (**a**–**d**) B3, (**e**–**i**) B8, and (**g**–**o**) B9 electrodes.

X-ray photoelectron spectroscopy (XPS) provides a valuable complementary technique for determining oxidation states and stoichiometry of oxides. The oxidation specificity of the released electron energy makes it easier to assign the identified bands. The binding energies of different peaks for the constructed B3, B8, and B9 electrodes are shown in Fig. 3. The XPS spectra revealed several oxidation states for Ru, Ti, Ir, and Ta, as shown in Table 3. The O 1 s spectra with lower binding energies (530.3–531.1 eV) correspond to the oxygen integrated into the MMO lattice (lattice oxygen O_lat_). In contrast, O 1 s spectra with higher energies (531.6–532.5 eV) are ascribed to adsorbed oxygen species (adsorbed oxygen O_ad_), such as hydroxide. The O_ad_/O_lat_ ratio indicates the electrochemical activity of the coated sauce, such that the electrochemical activity increases with a decrease in the O_ad_/O_lat_ ratio^24^. Ru^4+^ (286 eV) and Ru^2+^ (284 eV), which correspond to RuO_2_ and RuO, are detected in the Ru 3d spectra of all produced electrodes. Similarly, Ti 2p peaks are associated with Ti^4+^, Ti^3+^, and Ti^2+^ at (459, 457, and 460 eV) for TiO_2_, Ti_2_O_3_, and TiO, respectively. The oxidation state of Ir^4+^ for the matching IrO_2_ is connected to the peaks of the Ir 4f spectra (63 eV)^25^. Furthermore, the peaks near (28 eV) in the Ta 4f spectra are related to Ta^5+^ for the corresponding Ta_2_O_5_^26^. All XPS results show that the dominating oxide phases are RuO_2_, TiO_2_, IrO_2_, and Ta_2_O_5_, with the presence of minor additional Ru and Ti oxides.

**Table 3:** XPS analysis on the surface of B3, B8, and B9 electrodes.

Electrode	Binding energy (ev)/Atomic %					O 1s (lat) %	O 1s (ad) %	O_ad_/O_lat_
	O 1s	Ru 3d	Ti 2p	Ir 4f	Ta 4f			
B3	531.55/79.69	284.82/10.80	461.95/18.51	–	–	21.61	26.77	1.24
B8	531.82/59.42	284.94/15.36	462.88/17.52	63.53/7.70	–	35.12	41.45	1.18
B9	532.10/75.40	284.93/7.18	461.73/11.95	63.37/3.50	27.76/1.97	11.84	11.97	1.01

**Figure 4:**
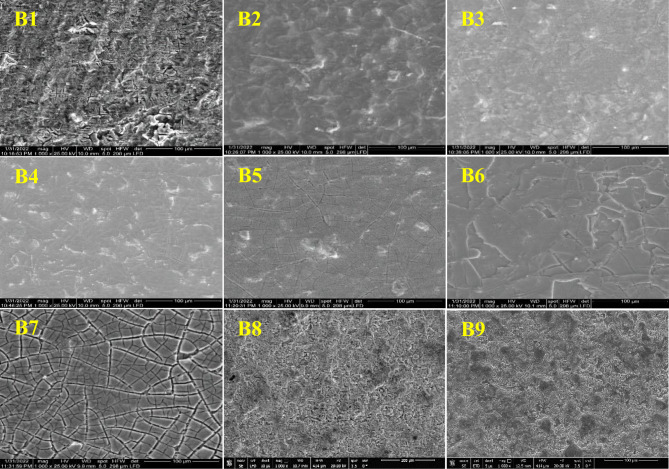
FESEM micrographs of the prepared (B1–B9) Ti/MMO samples.

FESEM micrographs of the prepared MMO coatings are shown in Fig. 4. At the lowest precursor concentration (0.01 M) for sample B1, the surface morphology appears to be tenuous. Generally, upon increasing the precursor concentration from 0.05 M up to 0.3 M for samples B2 to B5, the surface morphology improved, and the coating became more compact, fully covering the substrate. Further, increase in the precursor concentration to 0.4 M for sample B6, the coating developed into denser and overlapped layers. At the highest precursor concentration of 0.5 M for sample B7, the deposited layer turned into a cracked structure. The incorporation of IrO_2_ in sample B8 led to the formation of a coating with a fine spongy structure containing many pores^27^. The presence of Ta_2_O_5_ with IrO_2_ in sample B9 produced a more sealed coating in comparison to B8.

**Figure 5:**
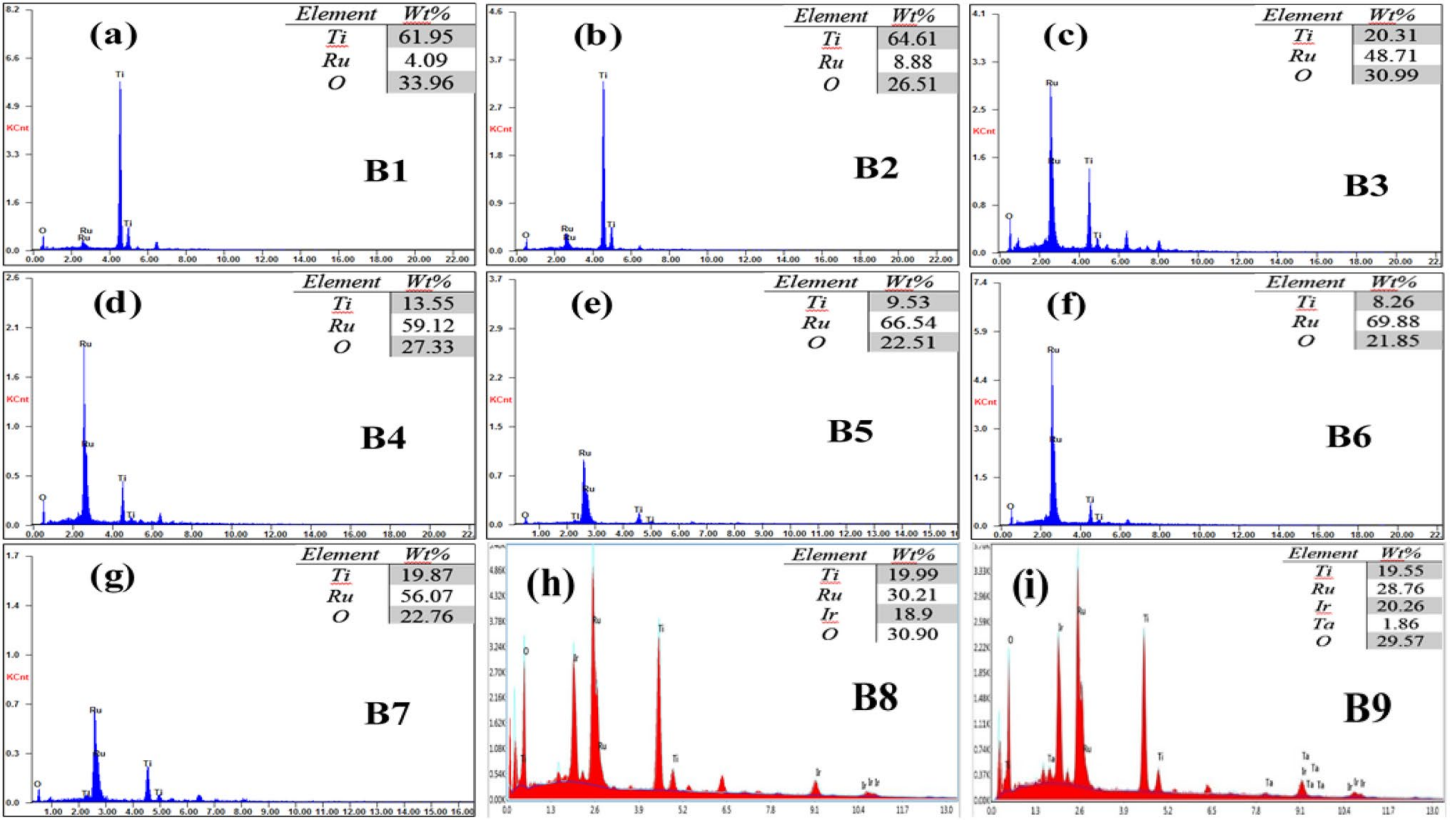
EDS analysis of the prepared (B1–B9) Ti/MMO samples.

Figure 5 shows the EDS point analysis of the prepared samples. As can be seen from the wt.% ratios, Ru, Ti, Ir, and Ta metallic elements were all converted into the corresponding oxides RuO_2_, TiO_2_, IrO_2_, and Ta_2_O_5_, as also disclosed from the XRD results. In the coating of sample B3 (Fig. 5c), the wt.% of Ru was found to increase rapidly, and that of Ti decreased as compared to the situations of both B1 and B2 samples (Fig. 5a and b). For samples B4, B5, and B6 (Fig. 5d–f)), the Ti wt.% slowly decreased, while the Ru wt.% attained almost similar values. Starting from B7 (Fig. 5g) to B9 (Fig. 5i) samples, the Ti wt.% achieved an average constant ratio very similar to that of sample B3 (Fig. 5c). Accordingly, the lowest concentration of RuCl_3_.3H_2_O and TiCl_3_ precursors that gave the almost desired MMO coating composition, is that of sample B3 (0.1 M). Concerning sample B9 (Fig. 5i), the resultant increase in the Ir ratio by 1.36 wt.% over that for sample B8 (Fig. 5h), is nearly equal to the decrease in the Ru ratio of sample B9 (1.45%) relative to that in sample B8. This confirms the synergistic influence of Ta on the rapid precipitation of Ir from the blend solution in the coating bath and at the same time, its hindrance effect on the Ru element precipitation.

### Thermal characterization

**Figure 6:**
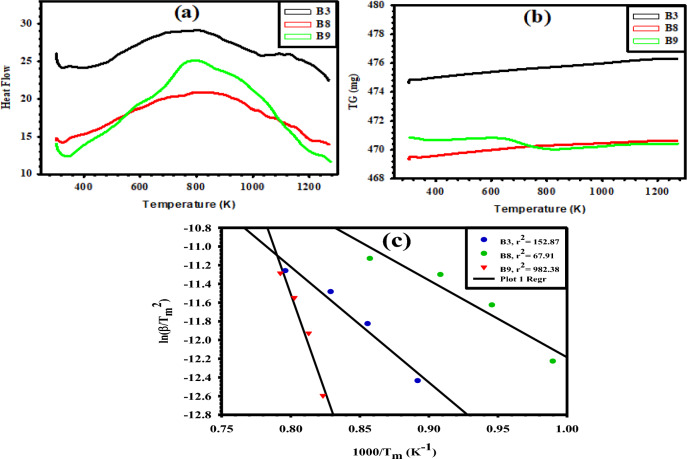
(**a**) DSC, (**b**) TG patterns at a heating rate of 10 K/min, and (**c**) Kissinger plots of the prepared B3, B8, and B9 MMO samples.

Differential scanning calorimetry (DSC) tests at various heating rates of 5, 10, 15, and 20 °C/min enable us to examine the kinetic parameters of the thermal reactivity and stability of our selected samples B3, B8, and B9 MMO coatings. The Kissinger method was used to estimate the activation energy (${{\text{E}}}_{{\text{a}}}$) by studying the shift in the DSC peak temperature caused by varying the heating rate^28,29^. The DSC curves for the coating samples at a constant heating rate of 10 °C/min are shown in Fig. 6a. The DSC results are listed in Table 4. The DSC curves at the other heating rates exhibited similar behavior. As shown in Fig. 6a, there are some endothermic peaks obtained in the temperature range higher than 1000 K for samples B3 (60%RuO_2_-40%TiO_2_), B8 (60%RuO_2_-30%TiO_2_-10%IrO_2_), and B9 (60%RuO_2_-20%TiO_2_-15%IrO_2_-5% Ta_2_O_5_). The thermogravimetry (TG) curves for the coating layers of samples B3, B8, and B9 are shown in Fig. 6b. Note that TG curves would not deliver any information about the temperature decomposition of those materials since the prepared MMO coating layers have already been subjected to a thermal treatment round.

**Table 4:** DSC results of the prepared MMO samples and their ${{\text{E}}}_{{\text{a}}}$ values.

Sample	Heating rate β (K/min)	T_m_ (K)	E_a_ (kJ/mol)
B3	5	1121.24	102.80
	10	1168.77	
	15	1206.38	
	20	1254.36	
B8	5	1010.23	68.51
	10	1057.57	
	15	1100.87	
	20	1166.45	
B9	5	1214.67	260.58
	10	1230.02	
	15	1245.88	
	20	1261.89	

The apparent activation energy (${{\text{E}}}_{{\text{a}}}$) for the produced MMO deposited coatings was evaluated by applying the Kissinger method on the first endothermic peak (T_m_), as stated in Eq. (11), which is particularly significant as it indicates the crystallization temperature:

11 $${\text{ln}}(\frac{\upbeta }{{{\text{T}}}_{{\text{m}}}^{2}})={\text{ln}}\left(\frac{{\text{AR}}}{{{\text{E}}}_{{\text{a}}}}\right)-\frac{{{\text{E}}}_{{\text{a}}}}{{{\text{RT}}}_{{\text{m}}}}$$

In this equation, β denotes the heating rate in K/min, T_m_ is the peak temperature in K, R is the universal gas constant (8.314 J/mol/K), ${{\text{E}}}_{{\text{a}}}$ is the apparent activation energy in J/mol, and A is the frequency factor in s^−1^. The ${E}_{{\text{a}}}$ value can be simply calculated from the slope of the linear Kissinger relationship ln (β/T_m_^2^) vs. 1/T_m_^30^, and multiplying its value by minus the universal gas constant (Eq. (12)), as illustrated in Fig. 6c, and listed in Table 4:

12 $${{\text{E}}}_{{\text{a}}}=-{\text{R}}\left\{\frac{{\text{d}}\left[{\text{ln}}\left(\frac{\upbeta }{{{\text{T}}}_{\mathrm{m }}^{2}}\right)\right]}{{\text{d}}\left(\frac{1}{{{\text{T}}}_{{\text{m}}}}\right)}\right\}$$

It is obvious from the obtained values depicted in Table 4 that, ${{\text{E}}}_{{\text{a}}}$ of sample B3 decreased from 102.80 kJ/mol to a lower value of 68.51 kJ/mol upon substituting IrO_2_ for 10% TiO_2_ in sample B8. In the meantime, ${E}_{{\text{a}}}$ increased by more than twice its value, achieving 260.58 kJ/mol, upon substituting 15% IrO_2_ and 5% Ta_2_O_5_ for 20% TiO_2_ in sample B9. The lower activation energy is due to possible reasons such as (i) crystalline lattice strain experienced during the heat treatment step, (ii) formation of smaller crystallite size, and (iii) enhanced particle size distribution^29^. According to the basics of chemical kinetics, the lower the activation energy barrier is, the higher the activity of the material to initiate a reaction and vice versa^31,32^. Thus, the B8 sample would be better than B3 as a catalyst for the CER and OER, while the B9 anode would be with lesser efficacy despite its higher stability, as will be seen in the coming sections. The tendency of the catalytic activity for the designed electrodes can thus be ordered as B8 > B3 > B9 according to the Kissinger analysis. This rank is in congruence with the SEM images shown in Fig. 3, as coatings of samples B3, B8, and B9 have different structural morphologies. Sample B8 has an exceptionally spongy-like structure with fine particles and many pores. This would be conducive to more active sites on its surface compared to those for samples B3 and B9. Also, compared with B8 and B3 samples the XRD pattern of sample B9 has a series of broad and low-intensity peaks (Fig. 2). This could be attributed to the presence of an amorphous phase in the coating and refinement in its structure^33^.

### Electrochemical characterization

#### Behavior in acid solution

**Figure 7:**
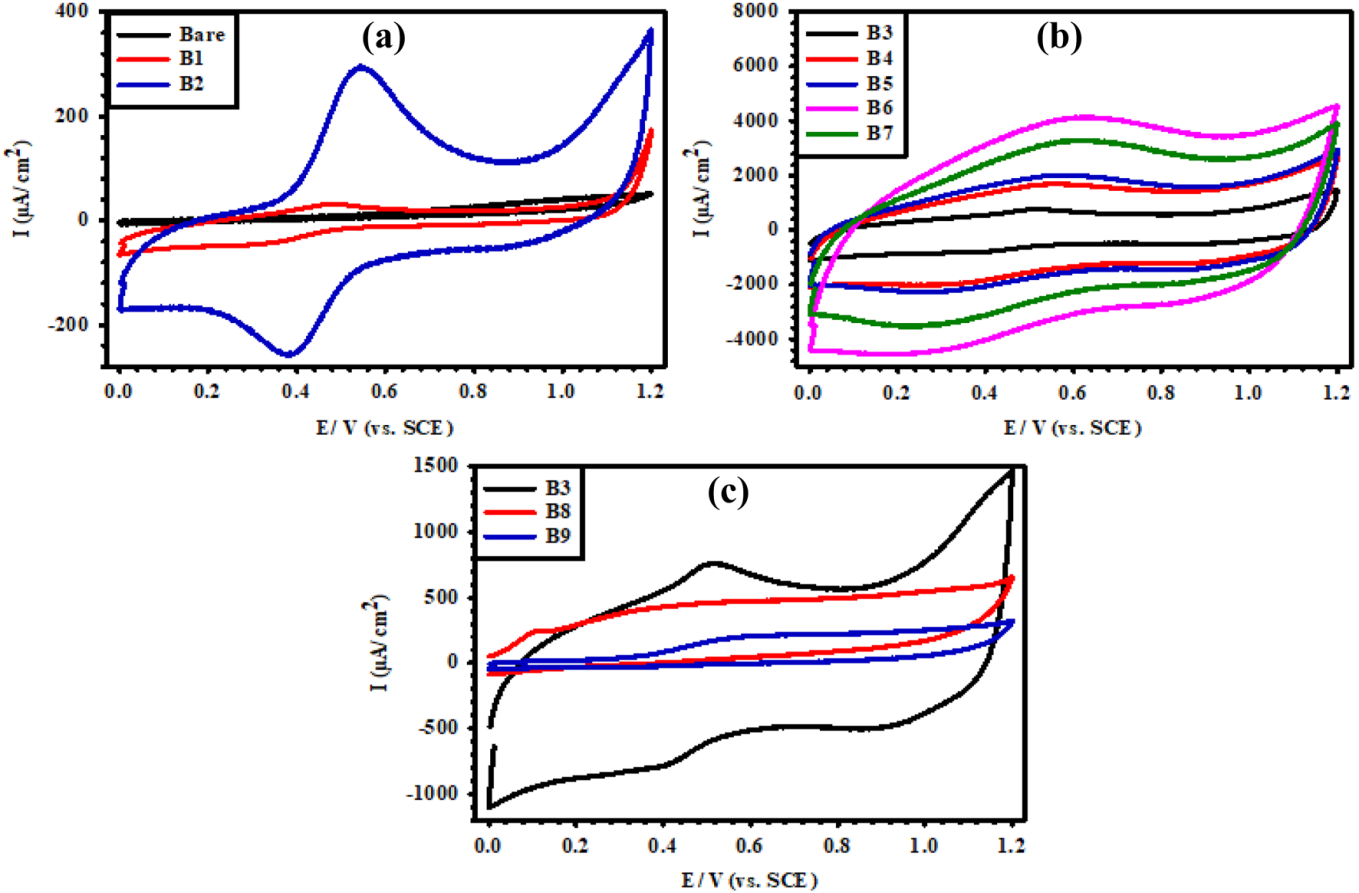
CV tests at a scan rate of 50 mV/s for the prepared (B1–B9) Ti/MMO electrodes in 1 M H_2_SO_4_ solution.

CV tests of the various coated electrodes were performed in 1 M H_2_SO_4_ solution over a potential window of 0 V to 1.2 V (vs. SCE) to investigate the properties of the coating surfaces, as shown in Fig. 7. In all cases, the forward scan revealed an anodic peak in the potential range between 0.5 V and 0.6 V associated with the Ru^2+^/Ru^3+^ redox reaction^34^. The corresponding cathodic peaks in the reverse scan appeared at a potential of 0.4 V with a shift of about 0.2 V, resulting from the unavoidable overpotential due to the strong interaction between the coating components and the oxide layer formed in the forward anodic scan^35,36^. Moreover, the similarity of both the anodic and cathodic profiles indicates the reversibility features of the redox reactions occurring on the Ti/MMO electrodes. The variation in the voltammograms of the different electrodes is linked to the variation in their compositions and microstructures, which leads to the modification in the active surface area. It should be noted that the trend of current observed in the CV curves of Fig. 7a,b is to increase with increasing the RuO_2_ content in the Ti/MMO electrodes^11^, in agreement with the results of EDS depicted in Fig. 5. The incorporation of IrO_2_ alone (electrode B8) and IrO_2_ with Ta_2_O_5_ (electrode B9) in the MMO coatings is conducive to a pronounced decrease in the measured current response as shown in Fig. 7c. The mixtures of these oxides with TiO_2_ exhibit an insulating behavior that impedes the activity of RuO_2_. In the meantime, they can enhance the corrosion resistance of the MMO coatings, thus enabling Ti/MMO electrodes to work efficiently under sustainable oxygen evolution in such a low-pH medium^37^.

**Figure 8:**
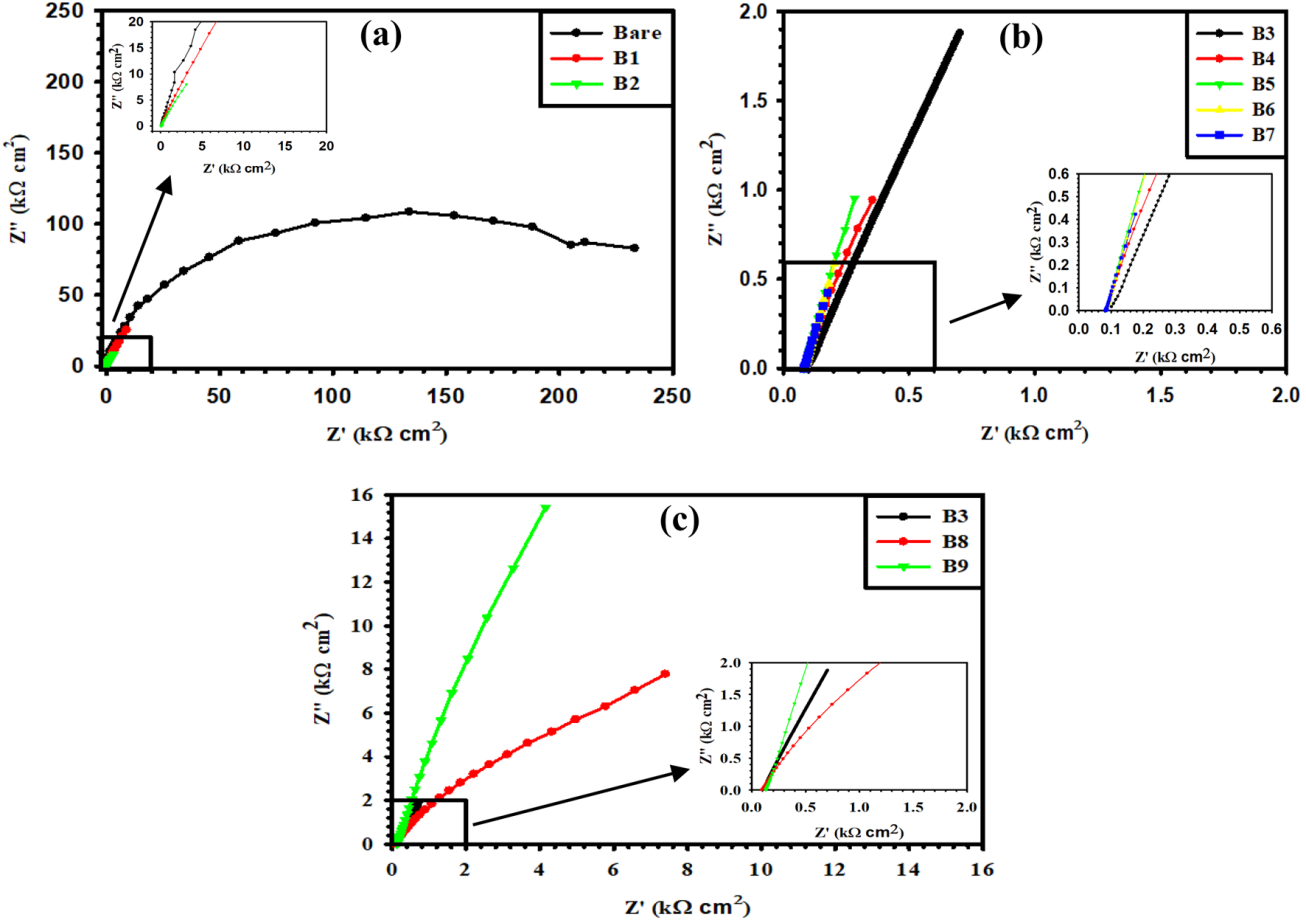
Nyquist diagrams of the prepared (B1–B9) Ti/MMO electrodes in 1 M H_2_SO_4_ solution.

**Figure 9:**
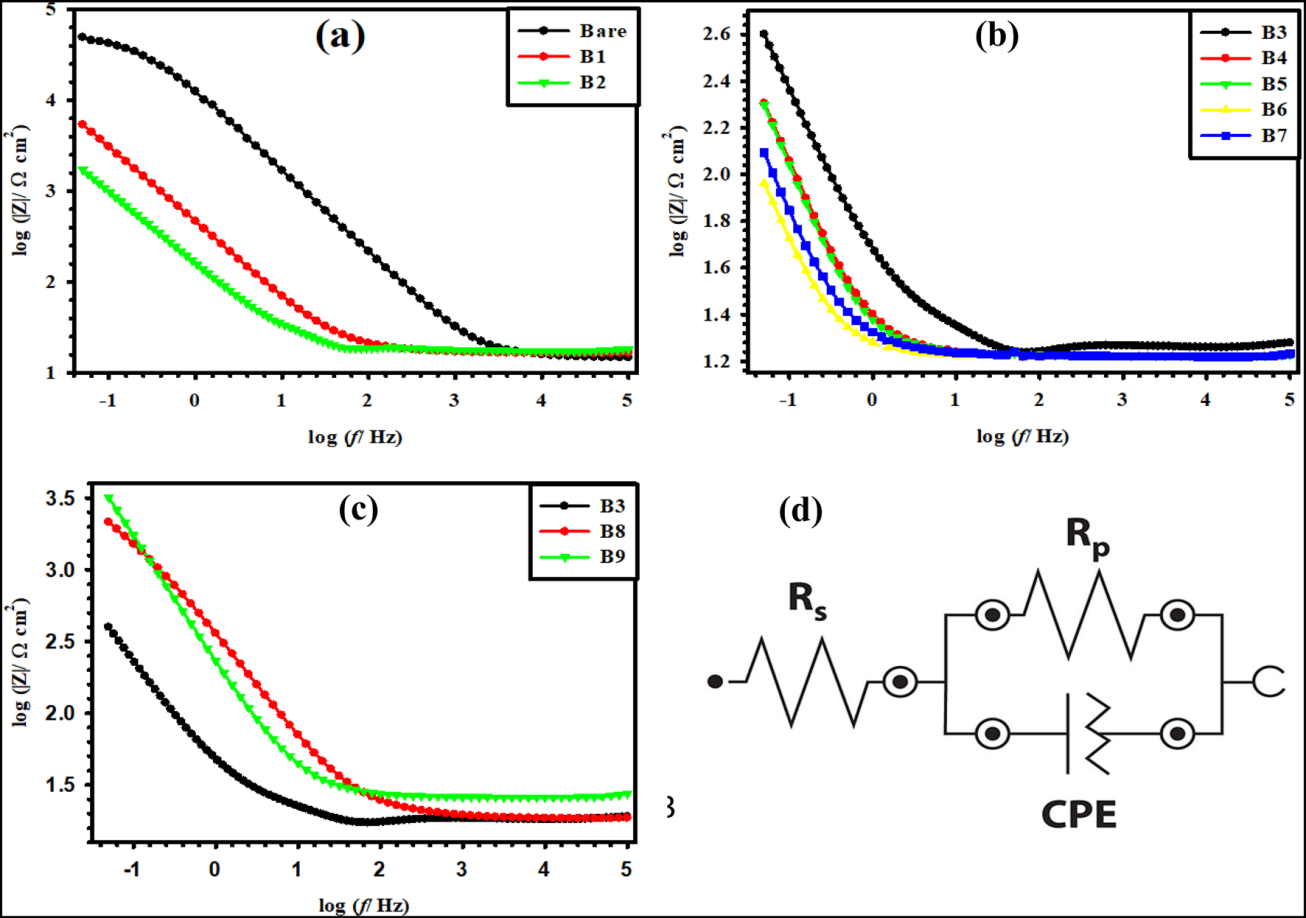
(**a**–**c**) Bode diagrams of the prepared (B1–B9) Ti/MMO electrodes in 1 M H_2_SO_4_ solution. (**d**) Equivalent circuit (EC) for fitting the measured EIS data.

To further scrutinize the interface properties of our synthesized binary, ternary, and quaternary Ti/MMO electrodes in a non-destructive manner, electrochemical impedance spectroscopy (EIS) was recorded in aqueous 1 M H_2_SO_4_ solution over a wide frequency range from 100 kHz down to 50 mHz using a sinusoidal perturbation signal with a low amplitude of 10 mV, as shown in Figs. 8 and 9. On the Nyquist format, the bare Ti surface presents an extended depressed semi-circle (Fig. 8a), which turns out to be a straight line with different slopes for the various coated samples (Fig. 8a–c). This behavior indicates smaller polarization resistance (*R*_p_) for the coated electrodes relative to that for the uncoated bare Ti electrode, albeit with different degrees depending on their compositions. Also, on the Bode format of the impedance modulus (|Z|) as a function of the frequency (*f*), Fig. 9a–c shows that despite the measurements being prolonged down to a very low f value of 50 mHz, all log (|Z|) vs. log (*f*) plots exhibit only one horizontal segment at the high-frequency range between 10^2^ and 10^5^ Hz. This segment with a zero slope matches the solution resistance (*R*_s_) between the working and reference electrodes^38,39^. However, at the medium and low frequencies, all plots present linear diagonals with almost similar slopes of (~ − 1). Such a behavior is typical for all metallic materials characterized by extremely low corrosion rates owing to their ability to form a highly resistive thin oxide film on their surfaces^40,41^. The results thus reflect the relatively high stability of our coated MMO sample in an acidic environment with a very low pH value.

The experimental EIS data were fitted to the most suitable equivalent circuit (EC) with a single time constant as depicted in Fig. 9d and the fitted values are given in Table 5. The *R*_p_ of the uncoated Ti sample is almost 10.72 kΩ cm^2^, while the *R*_p_ of the Ti/MMO coated electrodes is generally decreased with the increase in RuO_2_ content. The *R*_p_ values obtained are 5.22, 5.20, 2.32, 1.67, 1.24, 0.60, and 0.59 kΩ cm^2^ for B1 to B7 samples, respectively. The *R*_p_ of the B9 electrode is 8.50 kΩ cm^2^ larger than those for samples B3 and B8 prepared based on the same precursor concentration (0.1 M), with estimated *R*_p_ values of 2.32 kΩ cm^2^ and 1.49 kΩ cm^2^, respectively. This trend goes in parallel with the obtained apparent activation energy (E_a_) values of these three electrodes. Furthermore, as the RuO_2_ concentration decreased, the capacitive region shifted toward a higher frequency range, revealing lower capacitance values as depicted in Table 5. These results reconcile with the CV data in that the lower electrical resistance of the coated electrodes greatly improves their electrocatalytic properties, as will be seen in the next part. Good fitting analysis of the measured EIS data was obtained by implementing a constant phase element (CPE) in the EC shown in Fig. 9d instead of a real capacitance (*C*) to better describe the heterogeneity in the microstructures of our synthesized Ti/MMO electrodes^31^.

**Table 5:** Fitted EIS parameters of the prepared Ti/MMO electrodes in 1 M H_2_SO_4_ solution.

Sample	R_s_ (Ω cm^2^)	R_p_ (kΩ cm^2^)	CPE (mF cm^−2^ s^n^)
Ti Bare	2.86	10.72	0.08
B1	3.28	5.22	0.20
B2	2.14	5.20	0.56
B3	4.36	2.32	1.38
B4	3.34	1.67	1.91
B5	3.48	1.24	2.57
B6	3.32	0.60	5.25
B7	3.38	0.59	5.35
B8	3.62	1.49	2.13
B9	5.16	8.50	0.38

### Behavior in brine solution

**Figure 10:**
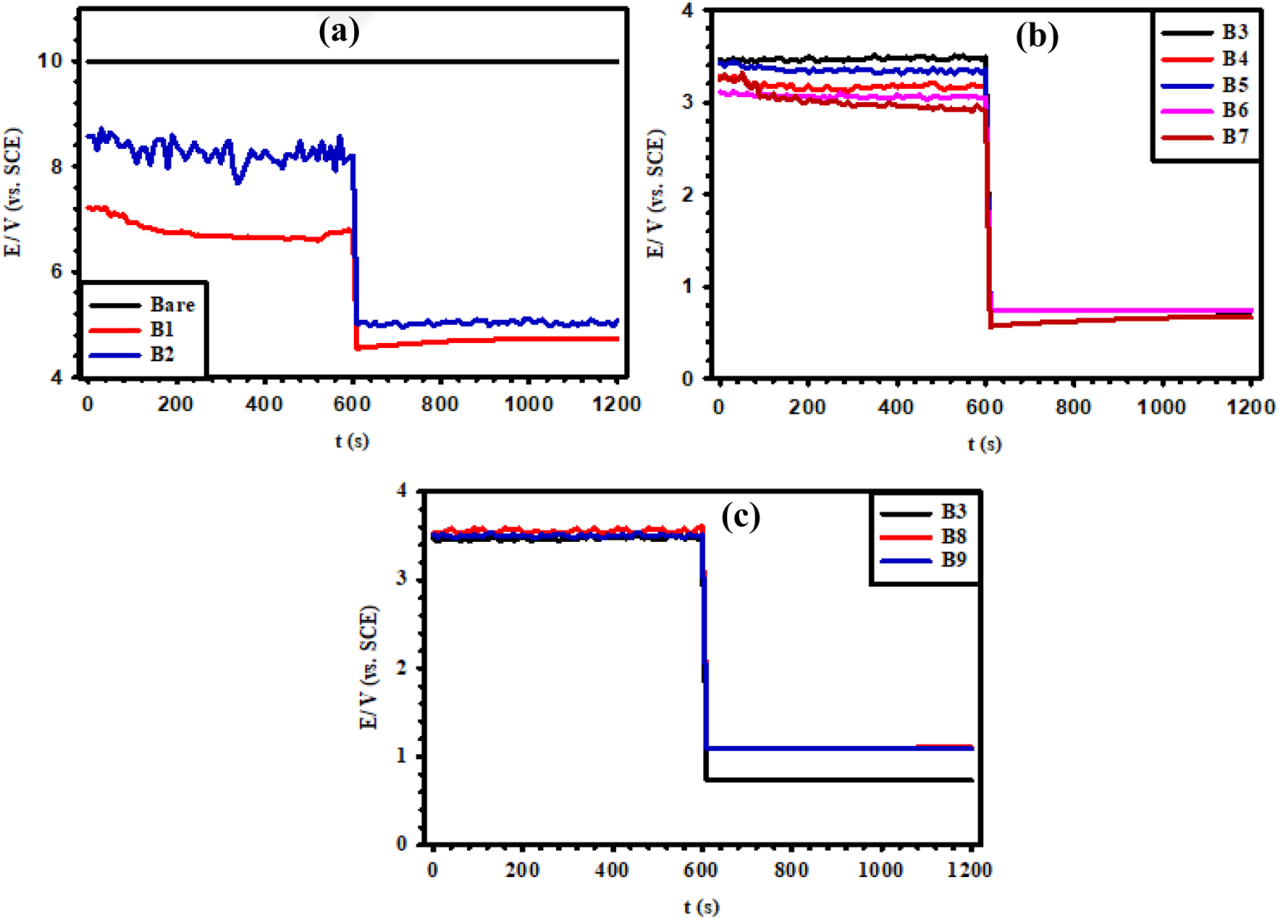
Current on–off test at 300 mA/cm^2^ for the prepared (B1–B9) Ti/MMO electrodes in (5 M NaCl + 0.01 M HCl) brine solution of pH 2.

The electrochemical activity of the synthesized Ti/MMO electrodes in (5 M NaCl + 0.01 M HCl) brine solution of pH 2 at room temperature was determined utilizing the current on–off test. As shown in Fig. 10, the MMO-coated electrode was activated by polarizing it at a current density of 300 mA/cm^2^ (3 kA/m^2^), which is the actual value used in the Chlor-alkali industry. The activation was extended for 10 min, being a sufficient time for activating the coated electrode surface and reaching a stable electrochemical state. At the end of minute 10, the current was interrupted, and the angle at the potential drop was measured. The final angle should be close to 90° to ensure proper MMO coating formation. As shown in Fig. 10b, B3 to B7 Ti/MMO electrodes acquired a stable potential ranging from 3.1 to 3.5 V (vs. SCE) during the current-on state. This is the potential range at which chlorine gas evolution is liable to occur. The angle formed following the potential drop upon current on–off was very close to 90°, indicating the formation of perfect coatings^42^. However, in Fig. 10a it is obvious that both B1 and B2 electrodes exhibit unstable electrochemical states. On the other hand, Fig. 10c reveals better performance for the two electrodes B8 and B9 as they both show a resultant angle near 90°. The presence of IrO_2_ gives almost the same potential in the active state and exhibits a straighter line without ridges, denoting improved efficiency. Additionally, the results disclose that the B9 electrode has superior stability than B8 or B3, confirming the importance of using not the ternary (RuO_2_-TiO_2_-IrO_2_) MMO, but better the quaternary (RuO_2_-TiO_2_-IrO_2_- Ta_2_O_5_) MMO to enhance both the efficiency and stability of the Ti/MMO electrode during CER from such aggressive brine solution^33^.

**Figure 11:**
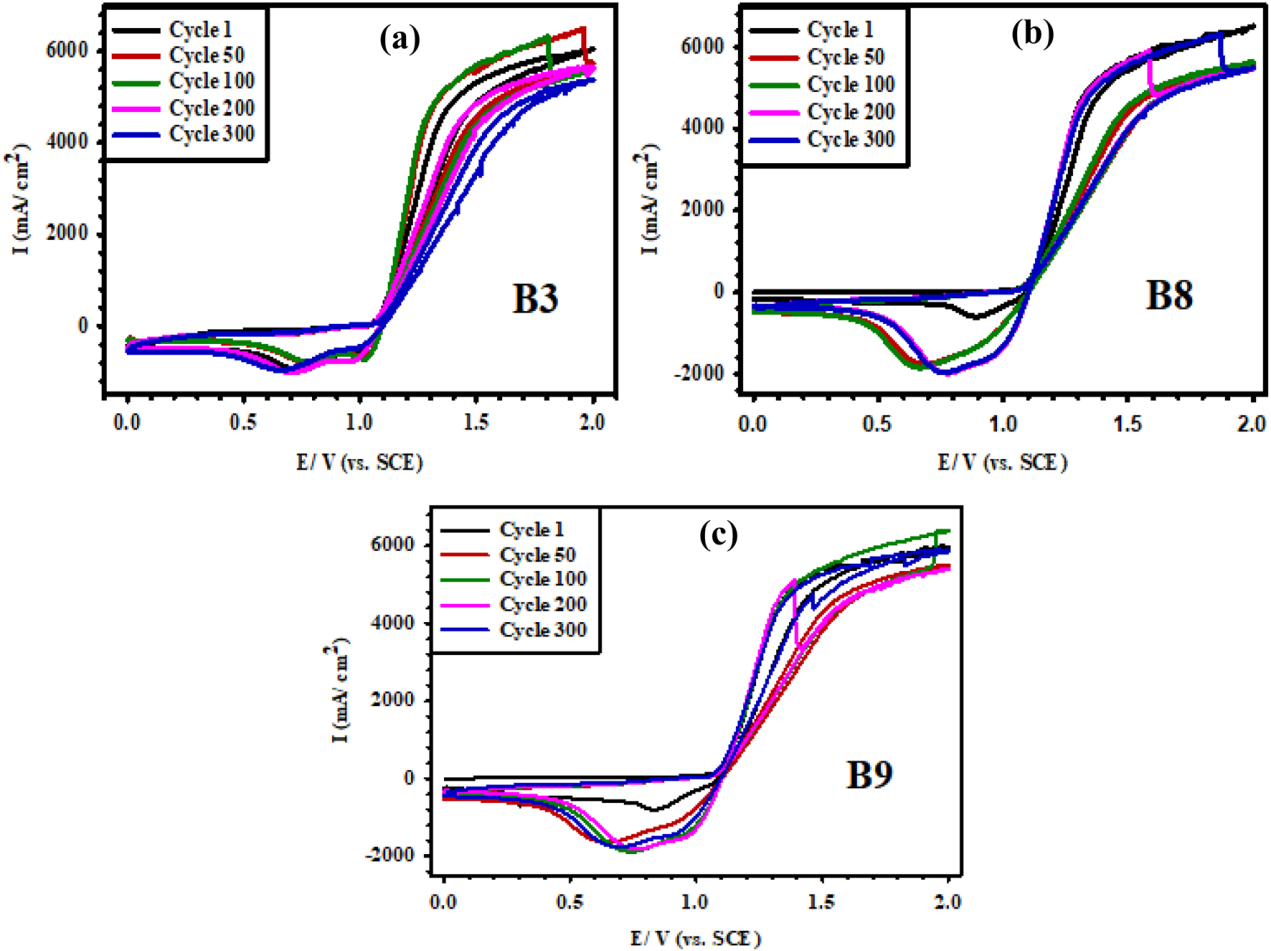
Cyclic voltammetry stability test (ST) at a scan rate of 50 mV/s over 300 cycles for the prepared B3, B8, and B9 Ti/MMO electrodes in (5 M NaCl + 0.01 M HCl) brine solution of pH 2.

Furthermore, CV measurements within the potential range of 0.0–2.0 V (vs. SCE) at a scan rate of 50 mV/s were used to test the stability of the synthesized MMO electrodes over 300 CV cycles in (5 M NaCl + 0.01 M HCl) brine solution of pH 2. The three electrodes B3, B8, and B9 prepared based on the same precursor concentration of 0.1 M with varying compositions, were selected to perform the stability tests. The 0.1 M is the lowest concentration from the precursors that produced properly coated electrodes. Figure 11 presents the CV curves for the three tested electrodes after the 1st, 50th, 100th, 200th, and 300th complete CV cycles. Overall, the stability of the three electrodes is quite good, yet for the B3 electrode, the current response experiences a slight decrease by cycling with a small gradual shift towards a lower potential value. Electrode B9 almost has the highest relative stability compared to B3 and B8 electrodes. This result goes in agreement with the trend of ${{\text{E}}}_{{\text{a}}}$ and R_p_ values found to be in the order B9 > B3 > B8. It is thus evident that the stability of the Ti/MMO electrodes in brine solution is a function of its composition such that the quaternary electrode containing IrO_2_ and Ta_2_O_5_ would outperform the ternary electrode containing IrO_2_ only or the basic binary (RuO_2_-TiO_2_) MMO electrode^33^.

**Figure 12:**
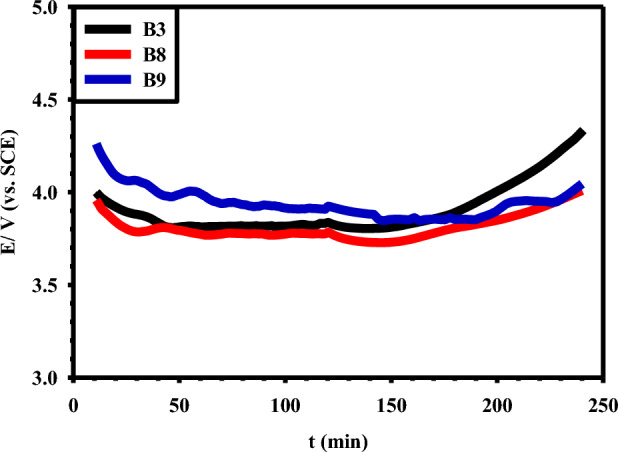
Accelerated stability test (AST) at a current density of 1 A/cm^2^ for the prepared B3, B8, and B9 Ti/MMO electrodes in (5 M NaCl + 0.01 M HCl) brine solution of pH 2.

The stability of the prepared electrodes was also assessed using chronopotentiometry via an accelerated stability test (AST) in (5 M NaCl + 0.01 M HCl) brine solution of pH 2 at a current density of 1 A/cm^2^, as shown in Fig. 12. The absence of any rapid breakdown in the potential of these anodes demonstrates their remarkable stability. The observed initial gradual decrease in the potential during the first 20–30 min for the three samples B3, B8, and B9 can be mainly attributed to the partial dissolution of the less stable anodized film that formed on the pristine coating. The catalytic efficiency of the B3 electrode appears to be significantly stable over the testing time, which extended for 240 min. The main factor contributing to the stability of the B3 electrodes is the distribution of the RuO_2_-TiO_2_ coating on the Ti substrate and the good mechanical adhesion properties between the coating and the Ti substrate, encouraged by the hydrothermal treatment^43^. The presence of IrO_2_, which prevents potential breakdown with time, gives the B8 electrode excellent corrosion resistance. The presence of both IrO_2_ and Ta_2_O_5_ in the B9 coating enhances further the stability of the electrode^44^. As expected, the steady-state potential (E_ss_ in V vs. SCE) follows the sequence B9 (3.96) > B3 (3.84) > B8 (3.76), which demonstrates the outperform stability of the sample B9 containing Ta_2_O_5_ in its MMO coating^33^.

### The CER and OER performance

**Figure 13:**
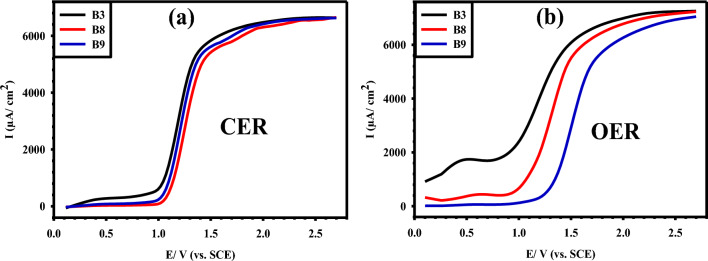
LSV curves of B3, B8, and B9 Ti/MMO electrodes at a sweep rate of 50 mV/s for: (**a**) CER in (5 M NaCl + 0.01 M HCl) brine solution of pH 2; and (**b**) OER in 1 M H_2_SO_4_ solution.

As indicated before the anodic chlorine evolution reaction (CER) process is accompanied by a slight oxygen evolution reaction (OER). Linear sweep voltammetry (LSV) was used to evaluate the performance of the selected synthesized electrodes (B3, B8, and B9) for CER and OER in (5 M NaCl + 0.01 M HCl) brine solution of pH 2 and 1 M H_2_SO_4_ solution, respectively, at a scan rate of 50 mV/s. In the brine electrolyte, the sharp increase in current starts at about 1.05 V for the B3 Ti/MMO anode, as shown in Fig. 13a, which is typically the potential of the chlorine gas evolution. The CER has a very small shift (1.07 and 1.06 V) after adding IrO_2_ or (IrO_2_ and Ta_2_O_5_), i.e. at B8 and B9 anodes, respectively, indicating approximately similar electrochemical reactivity towards the CER. On the other hand, in 1 M H_2_SO_4_ electrolyte, the potential at which the current jumps varied depending on the electrode composition, where it starts at 0.90 V for B3, 0.98 V for B8, and reaches 1.28 V for B9 anode, as shown in Fig. 13b. Under such conditions, the potential comprises both chlorine and oxygen gas evolutions. In both cases, the measured current density is higher in the B3 electrode compared to the B8 and B9 electrodes containing IrO_2_ and IrO_2_- Ta_2_O_5_. It is worth pointing out that the OER occurred before CER on the B3 and B8 electrodes. However, at the B9 electrode, CER occurred before OER and has an overpotential difference of 0.22 V between CER and OER, which indicates the important role of Ta_2_O_5_ implementation in inhibiting to some extent the OER. Several articles focused on improving catalytic efficiency against CER and OER^45–50^. Table 6 provides a summary of the enhanced onset potential of the CER and OER for various MMO electrodes.

**Table 6:** Summary of onset potential (*E*_on_ vs. SCE) of various anodes against CER and OER.

Anode	Electrolyte	CER (V)	OER (V)	*E*_on_ Shift compared with B3	Ref
Ru	0.1 M H_2_SO_4_, ν = 10 mV/s	–	0.96	0.06	^45^
Ir	0.1 M H_2_SO_4_, ν = 10 mV/s	–	1.03	0.13	^45^
RuO_2_	0.1 M H_2_SO_4_, ν = 10 mV/s	–	1.11	0.21	^45^
IrO_2_	0.1 M H_2_SO_4_, ν = 10 mV/s	–	1.23	0.33	^45^
TiO_2_-NTs/SnO_2_-Sb	0.5 M H_2_SO_4_, ν = 50 mV/s	–	1.95	1.05	^46^
Ti/SnO_2_-Sb	0.1 M Na_2_SO_4_, ν = 10 mV/s	–	1.81	0.91	^47^
TiO_2_-NTs/SnO_2_	0.1 M Na_2_SO_4_, ν = 100 mV/s	–	1.56	0.66	^48^
Ti/IrO_2_-RuO_2_	Saturated NaCl	1.10	–	0.05	^49^
Ti/IrO_2_-RuO_2_	0.5 M H_2_SO_4_	–	1.36	0.46	^49^
Ti/IrO_2_-Ta_2_O_5_	Saturated NaCl	1.11	–	0.06	^49^
Ti/IrO_2_-Ta_2_O_5_	0.5 M H_2_SO_4_	–	1.39	0.49	^49^
RuO_2_-TiO_2_/Ti (TR)	0.5 M NaCl, ν = 100 mV/s	1.12	–	0.07	^8^
La-doped RuO_2_-TiO_2_/Ti (TRL)	0.5 M NaCl, ν = 100 mV/s	1.08	–	0.03	^8^
(TR)	1 M H_2_SO_4_, ν = 100 mV/s	–	1.20	0.30	^8^
(TRL)	1 M H_2_SO_4_, ν = 100 mV/s	–	1.20	0.30	^8^
(Ti, Ru)O_2_	0.5 M NaCl, ν = 5 mV/s	0.29	–	− 0.76	^50^
(Ti, Ru)O_2_	1 M H_2_SO_4_, ν = 5 mV/s	–	0.57	− 0.33	^50^
(Ti, Ru, Ir)O_2_	0.5 M NaCl, ν = 5 mV/s	0.98	–	− 0.07	^50^
(Ti, Ru, Ir)O_2_	1 M H_2_SO_4_, ν = 5 mV/s	–	1.24	0.34	^50^
B3	(5 M NaCl + 0.01 M HCl) brine solution of pH 2, ν = 50 mV/s	1.05	–	–	–
B8		1.07	–	–	–
B9		1.06	–	–	–
B3	1 M H_2_SO_4_, ν = 50 mV/s	–	0.90	–	–
B8		–	0.98	–	–
B9		–	1.28	–	–

*ν* scan rate.

## Conclusions

Binary RuO_2_-TiO_2_ MMO coatings with various compositions (B1-B7) on Ti substrates were successfully synthesized via a simple green dipping method followed by heat treatment steps. Among the tested precursor concentration range used to prepare the various MMO binary coatings, 0.1 M (sample B3) was found to be the minimal concentration that gave the target weight gain. For that, this precursor dose was used for the preparation of the B8 ternary (RuO_2_-TiO_2_-IrO_2_) and B9 quaternary (RuO_2_-TiO_2_-IrO_2_ -TiO_2_) MMO samples. The sharp peaks in the XRD patterns of the acquired MMO coatings affirmed their crystalline nature, while their CV curves revealed a pair of pseudo-reversible peaks corresponding to the Ru^3+^/Ru^2+^ redox couple. The peak current response was found to increase with increasing RuO_2_ amount in the coating. The incorporation of IrO_2_ alone and IrO_2_ with Ta_2_O_5_ enhanced the electrochemical performance and stability of Ti/MMO electrodes. Relative to sample B3, the addition of IrO_2_ reduced its activation energy (${E}_{{\text{a}}}$) and polarization resistance (*R*_p_). OER occurs before CER with a small overpotential difference of 0.15 V and 0.09 V (vs. SCE) on the Ti/RuO_2_-TiO_2_ and Ti/RuO_2_-TiO_2_-IrO_2_ anodes, respectively. However, the addition of IrO_2_ with Ta_2_O_5_ in sample B9 was conducive to increasing both ${E}_{{\text{a}}}$ and *R*_p_ values of the quaternary MMO coating. In addition, the chlorine and oxygen evolution reactions occurred on this anode at 1.06 V and 1.28 V (vs. SCE), respectively, with a quite good overpotential separation of 0.22 V between them. Overall, the present work demonstrates the superiority of the proposed novel quaternary MMO coating anode (Ti/RuO_2_-TiO_2_-IrO_2_-Ta_2_O_5_) in electrochemical engineering applications such as the important chloro-alkali industries. This could be verified based on its high corrosion resistance, electrochemical stability, and long-life operating cyclability.

### Supplementary Information

Supplementary Figure S1.

## Data Availability

Data and materials will be available upon request from the corresponding author F. El-Taib Heakal.
