# Supplementary material for: Green synthesis and characterization of binary, ternary, and quaternary Ti/MMO anodes for chlorine and oxygen evolution reactions
Source: Sci Rep. 2024 Apr 29;14:9821. doi: 10.1038/s41598-024-59595-2 (PMC11058822; doi:10.1038/s41598-024-59595-2)
Supplement: Supplementary file 1 — Supplementary Figure S1. [file 41598_2024_59595_MOESM1_ESM.docx]

**Green synthesis and characterization of binary, ternary, and quaternary Ti/MMO anodes for Chlorine and Oxygen Evolution Reactions**

**A.B. Abdel-Aziz^1^, F.El-Taib Heakal^2*^, R.M. El Nashar^2^, I.M. Ghayad^3**^**

*^1^October High Institute for Engineering & Technology, 12596 Giza, Egypt.*

*^2^Chemistry Department, Faculty of Science, Cairo University, 12613 Giza, Egypt.*

*^3^Central Metallurgical Research and Development Institute (CMRDI), Cairo 12422, Egypt.*

********Correspondence author: Email:* [*hfakiha@cu.edu.eg*](mailto:hfakiha@cu.edu.eg) *(F. El-Taib Heakal)*

*Email*: [*ighayad@yahoo.com*](mailto:ighayad@yahoo.com) *(I.M. Ghayad)*

**Figure S1.** Cyclic voltammograms of two quaternary coated electrodes with two different Ta_2_O_5_: 5% (black) and 10% (red) in 1 M H_2_SO_4_ solution at a scan rate of 10 mV/s.
